# Platelet Count Recovery after Endovascular Aneurysm Repair for Abdominal Aortic Aneurysm

**DOI:** 10.3400/avd.oa.20-00030

**Published:** 2021-03-25

**Authors:** Kentaro Inoue, Tadashi Furuyama, Shun Kurose, Shinichiro Yoshino, Ken Nakayama, Sho Yamashita, Koichi Morisaki, Masaki Mori

**Affiliations:** 1 Department of Surgery and Science, Graduate School of Medical Sciences, Kyushu University, Fukuoka, Fukuoka, Japan; 2 Department of Vascular Surgery, Beppu Medical Center, Beppu, Oita, Japan

**Keywords:** abdominal aortic aneurysm, platelet, endovascular aneurysm repair, endoleak, aneurysm sac expansion

## Abstract

**Objective:** To find a new predictor of endoleak (EL) and aneurysm sac expansion after endovascular aneurysm repair (EVAR), we evaluated the platelet count recovery (PCR) process after EVAR.

**Materials and Methods:** Two hundred five patients treated with elective EVAR from 2007 to 2015 were retrospectively analyzed. We compared the platelet count ratio until postoperative day (POD) 7 to the presurgical baseline between patients with and without persistent EL (≥ 6 months). Subsequently, we calculated the optimal platelet count ratio for distinguishing persistent EL using receiver-operating characteristics analysis. A platelet count ratio on POD7 ≥118% was defined as the PCR. We evaluated the PCR’s influence on the cumulative aneurysm sac expansion rate.

**Results:** The average platelet count ratio on POD7 rose above baseline (112%), and the ratio was attenuated by persistent EL (103%). Of 205 patients, 126 (61%) were assigned to the disturbed PCR group (PCR(−) group). Cumulative aneurysm sac expansion rate was higher in the PCR(−) group than the PCR(+) group (34.4% vs. 12.8% in 5 years, p=0.01).

**Conclusion:** Disturbed PCR after EVAR may be associated with ELs and eventual aneurysm sac expansion.

## Introduction

Over the past 30 years, endovascular aneurysm repair (EVAR) has been widely used to treat abdominal aortic aneurysms (AAA). The procedure is minimally invasive and has demonstrated perioperative safety, even for high-risk patients. However, follow-up data has identified aneurysm sac expansion after EVAR as a significant complication in 25–40% of the cases.^1,2)^ Furthermore, a recent study suggested that aneurysm sac expansion is an independent risk factor for delayed mortality, requiring re-intervention after EVAR.^3)^ Postoperative aneurysm sac expansion is due primarily to persistent endoleak (EL). Type I or type III EL is regarded as a definitive indication for re-intervention. Further, even some type II ELs can contribute to sac expansion. Still, approximately half of EL cases resolve spontaneously.^4–8)^ Hence, careful follow-up and post-procedural examinations are as important as EVAR operative procedures to long-term patient outcomes.

Contrast-enhanced computed tomography (CT) and color duplex ultrasound imaging are the gold standards for follow-up examinations after EVAR.^9,10)^ These examinations only provide morphological information at that time, which is sometimes insufficient for clinical practice. For instance, detecting type II ELs at a certain point offers very little guidance about the indication for re-intervention because how the type II ELs behave in the future is unclear.^9,10)^ Furthermore, some AAAs can enlarge even in the absence of ELs.^11,12)^ Therefore, post-EVAR surveillance improves with another approach independent of morphological assessment.

Recently, Arnaoutoglou et al. suggested that EVAR causes platelet activation in the first 24 h post-surgery and then platelet count reduction over the next 72 h.^13)^ They also reported a correlation between postoperative platelet activation and total AAA volume, suggesting that platelet count may be predictive of AAA prognosis after EVAR. However, there are no studies on the association between platelet counts following EVAR and mid- and long-term clinical outcomes. In this study, we monitored the perioperative changes in platelet count and evaluated whether these changes predict the mid-term prognosis of AAAs following EVAR.

## Materials and Methods

### Study design and ethics approval

This retrospective study assessed patient outcomes using routine diagnostic and treatment data. The hospital institutional ethics committee approved this study, which was conducted under the Declaration of Helsinki (Approval No. 30-466). All patients provided written informed consent before treatment.

### Patients

Patients who underwent elective EVAR for non-ruptured AAA from September 2007 to December 2015 were evaluated retrospectively. The group consisted of 303 potential study candidates. We excluded cases with concomitant thoracic aortic aneurysm and/or iliac artery aneurysm to minimize the influence of non-AAA aneurysms on the platelet count. Consequently, we included 230 patients in this retrospective analysis. Patient information included age, sex, smoking history, comorbidities, medications, preoperative platelet count, postoperative platelet count (from postoperative day [POD] 1, POD3 and POD7), aneurysm characteristics (shape, size), treatment procedures, presence/absence of EL, and EL type and outcome (cumulative aneurysm sac expansion rate and overall survival).

### Laboratory data and platelet count recovery

All preoperative data, including complete blood count (CBC), were collected within one month before treatment. CBCs were also collected on POD1, POD3, and POD7. In this study, the ratio of postoperative platelet count to the platelet count observed before the operation was defined as the platelet count ratio as follows:
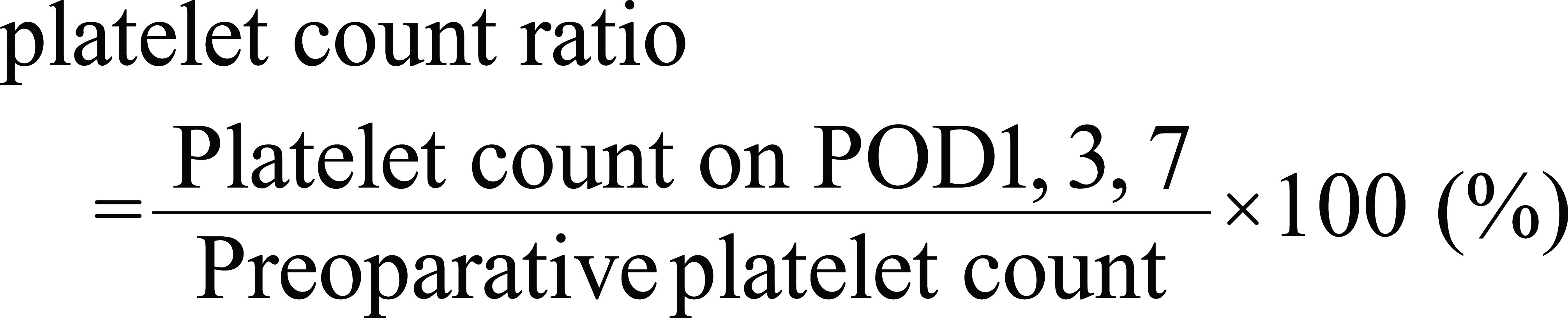


We used available data to calculate the platelet count ratios of patients with and without persistent EL to examine whether the EL could influence platelet count changes.

Next, we conducted a receiver-operating characteristics (ROC) analysis to identify a cut-off value of platelet count ratio on POD7 for high detection of ELs. The cut-off value was calculated with the best accuracy to distinguish persistent EL (≥ 6 months), and the cut-off value was defined as the ‘platelet count recovery (PCR)’ for further analysis.

### Aneurysms characteristics and treatment procedures

A CT scan revealed the aneurysm’s shape and size. We assessed the number and model of stent-grafts and the proximal and distal landing details to classify treatment procedures. In this study, we used the Zenith Flex® (Cook Inc., Indianapolis, IN, USA), Powerlink® (Endologix, Irvine, CA, USA), GORE® Excluder® (W. L. Gore & Associates, Flagstaff, AZ, USA), Endurant II® (Medtronic Inc., Santa Rosa, CA, USA) and AORFIX® (Lombard Medical Technologies, Oxfordshire, UK). All EVAR treatments were performed under general anesthesia with surgical cut-down femoral artery access.

### Follow-up and diagnosis of endoleak and aneurysm sac expansion

Contrast-enhanced biphasic CT examined patients during the first 7 postoperative days. Follow-up CT scans were also performed 1, 3, 6, and 12 months post-EVAR and then either biannually or annually after that. An EL lasting ≥6 months was classified as persistent and assessed as a risk factor for enlarged AAA. Patients were followed-up for mortality and aneurysmal sac growth over time. Aneurysm sac expansion was defined as an increment >5 mm over the maximum pre-EVAR axial diameter.^9,10)^ The mean follow-up period for all patients was 3.7±0.2 years [95% confidence interval (CI): 3.3–4.0 years].

For further analysis, ELs were classified into two groups as follows. Benign EL included type II ELs with spontaneous resolution or without sac expansion. In contrast, malignant ELs included primary and secondary type I or III ELs and type II ELs with sac expansion during observation.

### Midterm outcomes

Cumulative aneurysm sac expansion rate was evaluated as the period to sac growth >5 mm relative to pre-EVAR regardless of additional treatment. The overall survival rate was also assessed.

### Statistical analysis

All statistical analyses were performed using JMP ver.13 (SAS Institute Inc., Cary, NC, USA). Categorical variables are expressed as numbers or percentages and continuous variables as mean with standard error and [range]. The chi-squared test compared the categorical variables. Continuous variables were compared by t-test after confirming the normal distribution using the Shapiro–Wilk test. The ROC analysis calculated the platelet count ratio’s cut-off value on POD7 with the best accuracy to distinguish persistent EL (≥6 months). The Kaplan–Meier method estimated the cumulative aneurysm sac expansion and overall survival rates. We compared the resulting Kaplan–Meier curves using the log-rank test. A Cox proportional-hazards model was used for univariate and multivariate analyses of risk factors for cumulative aneurysm sac expansion. A p<0.05 (two-tailed) was considered statistically significant for all tests.

## Results

### Changes in platelet count ratio after EVAR

**Figure 1:**
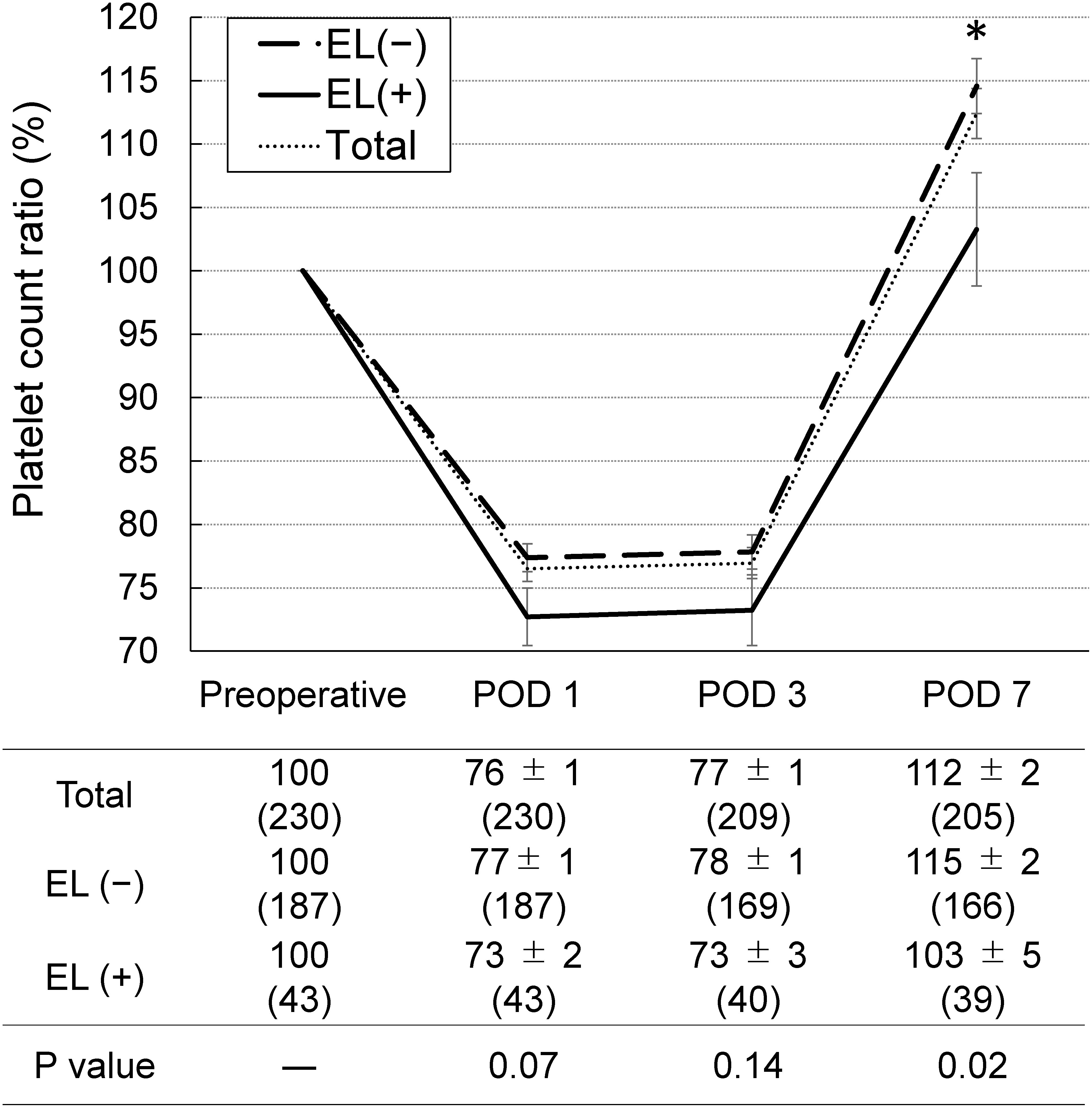
Fig. 1 Changes in platelet count ratio following endovascular aneurysm repair for abdominal aortic aneurysm. The number of patients is given in parentheses. Continuous data are shown as mean±standard error.

POD: postoperative day; EL: endoleak

We collected baseline platelet count data from all 230 cases (100%), retrieved platelet count data on POD1 and POD3 from 209 cases (91%), and collected data from baseline to POD7 from 205 cases (89%). All patients exhibited a 24% reduced platelet count ratio on POD1, and that reduction remained until POD3 (**Fig. 1**, dotted line). The average platelet count ratio rose until POD7 (112%±2%), with most of the study subjects (140/205, 68%) demonstrating this overshoot (>100%).

The patients were divided into two groups according to the presence or absence of persistent EL (all types) (**Fig. 1**) and compared platelet count values. The absolute preoperative platelet count did not differ between EL(+) and EL(−) groups (185±9×10^3^/µL [168–206] vs. 188±4×10^3^/µL [180–197]; p=0.74). Similarly, the magnitude of the transient platelet count dip during POD1 to POD3 did not differ. Alternatively, the magnitude of the overshoot on POD7 was larger in the EL(−) group than the EL(+) group (115%±2% vs. 103%±5%, p=0.02).

### Endoleak and the platelet count ratio on POD7

Within the first post-EVAR month, CT scans detected primary EL in 57 cases, including 3 cases of type I and 54 cases of type II. Of these type II cases, 15 of 54 (28%) disappeared spontaneously at 6 months or more post-EVAR, whereas 7 developed to type I (13%) and 3 to type III (6%). One case had a de novo secondary type II EL. Consequently, persistent EL (≥6 months) was recognized in 43 of 230 patients (20%) (10 type I cases, 30 type II cases, and three type III cases).

**Figure 2:**
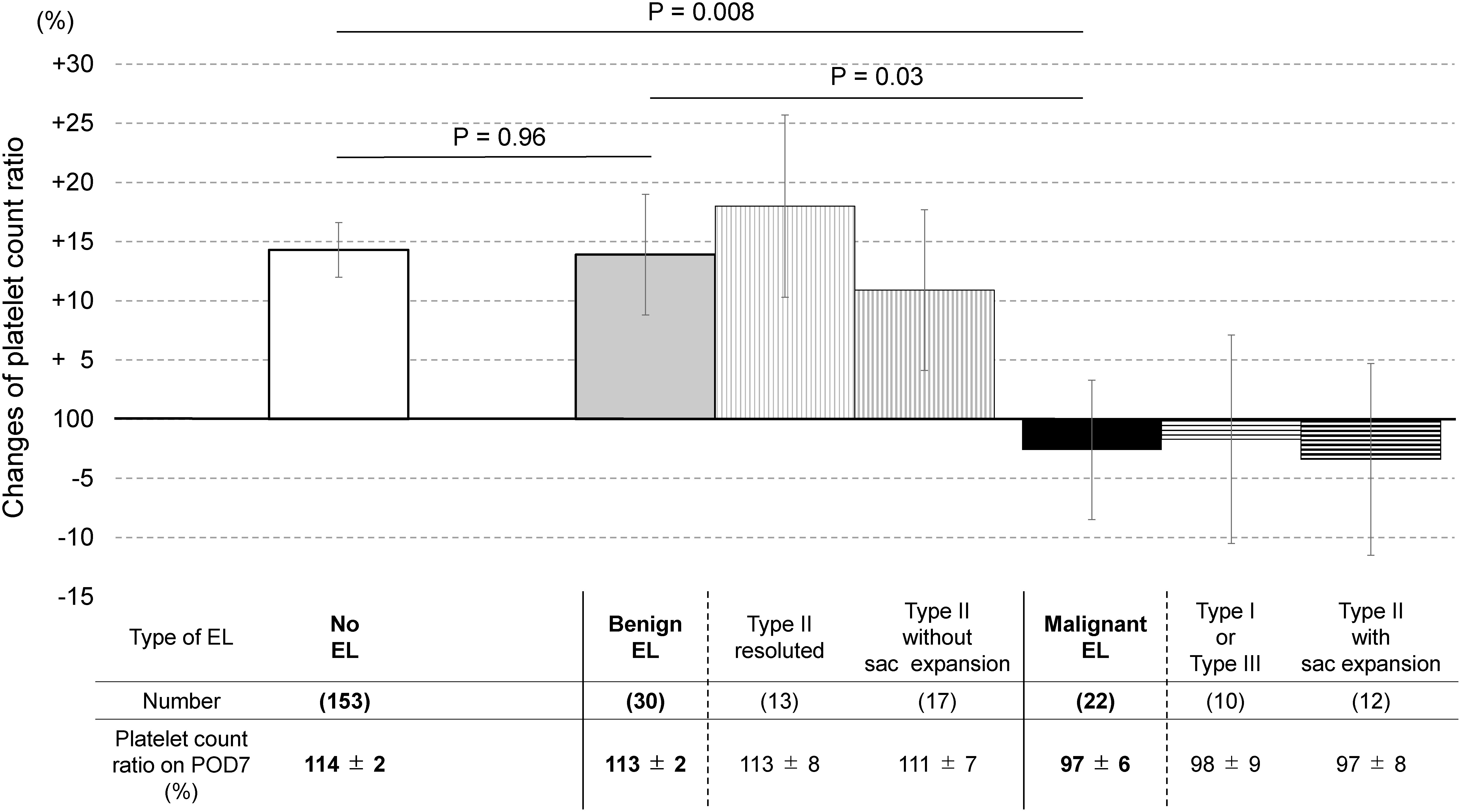
Fig. 2 Comparison of the platelet count ratio according to the type of endoleak (EL). The number of patients is given in parentheses. Continuous data are shown as mean±standard error. Data from one case of type-II EL with sac expansion, two cases of resolved type-II EL, and three cases of type-I or -III EL were excluded because of lack of laboratory data.

We compared the platelet count ratio on POD7 according to EL type (**Fig. 2**). Patients with no or only benign EL (type II with spontaneous resolution or without sac expansion) showed a recovery ratio above the baseline (>113%). Meanwhile, the platelet count of patients with “malignant” ELs (type I or type III or type II with sac expansion) could not recover the decrease (<100%).

We calculated the cut-off value with best accuracy to distinguish persistent EL (≥6 months), and the cut-off platelet count ratio (PCR) in our patients was 118% (area under the curve=0.62, sensitivity=79.5%, specificity=43.4%).

### Patient, aneurysm and treatment characteristics in groups with and without PCR

We then compared various characteristics between patients divided into a PCR(−) group (PCR <118%, N=126, 61% of the entire cohort with platelet count measures on POD7) and a PCR(+) group (PCR ≥118%). **Table 1** shows demographic and preoperative factors, and **Table 2** summarizes EVAR- and AAA-related parameters.

**Table 1:** Table 1 Patient characteristics and laboratory findings from AAA patient groups with and without optimal PCR*

Variables	Total (n=205)	PCR(+) (n=79)	PCR(−) (n=126)	P-value
Age (years)	75.0±0.5 (73.9–76.0)	73.8±0.9 (72.0–75.6)	76.1±0.7 (74.7–77.6)	0.05
Sex (male)	173 (84)	74 (94)	99 (79)	0.005
Body mass index	23.1±0.2 (22.6–23.5)	23.3±0.4 (22.4–24.2)	22.8±0.3 (22.1–23.5)	0.37
Smoking history				
Current	48 (24)	21 (27)	27 (22)	0.04
Past	56 (27)	14 (18)	42 (34)	
Never	100 (49)	44 (56)	56 (45)	
Hypertension	169 (82)	62 (78)	107 (85)	0.26
Diabetes mellitus	51 (25)	18 (23)	33 (26)	0.62
Dyslipidaemia	74 (36)	31 (39)	43 (34)	0.46
Coronary artery disease	65 (32)	21 (27)	44 (35)	0.22
Cerebrovascular disease	54 (26)	24 (30)	30 (24)	0.33
Ejection fraction (%)	65.4±0.8 (63.8–67.1)	65.0±1.4 (62.2–67.7)	65.9±1.1 (63.7–68.0)	0.60
FEV1.0%	68.6±0.8 (67.0–70.2)	67.5±1.4 (64.7–70.2)	69.8±1.1 (67.6–71.9)	0.19
Atrial fibrillation	18 (9)	5 (6)	13 (10)	0.45
End-stage renal disease	6 (3)	2 (3)	4 (3)	1.00
Medication				
Statin	105 (51)	45 (57)	60 (48)	0.20
Antiplatelet therapy				
None	99 (48)	36 (46)	63 (50)	0.57
SAPT	84 (41)	36 (46)	48 (38)	
DAPT	22 (11)	7 (9)	15 (12)	
Anticoagulant therapy	18 (9)	8 (10)	10 (8)	0.62
Laboratory data				
White blood cells (10^3^/µL)	6.6±0.1 (6.3–6.8)	6.6±0.2 (6.1–7.0)	6.5±0.2 (6.1–6.9)	0.79
Haemoglobin (g/dL)	12.8±0.1 (12.5–13.0)	12.8±0.2 (12.4–13.2)	12.7±0.2 (12.4–13.0)	0.55
Platelet count (10^3^/µL)	188±4 (180–195)	173±6 (160–186)	194±5 (183–204)	0.01
C-reactive protein (mg/dL)	0.75±0.01 (0.51–0.99)	0.73±0.2 (0.33–1.13)	0.70±0.16 (0.38–1.00)	0.88
Albumin (g/dL)	4.02±0.03 (3.96–4.09)	4.02±0.05 (3.91–4.13)	4.02±0.04 (3.94–4.11)	0.98
Creatinine (mg/dL)	1.14±0.06 (1.03–1.26)	1.20±0.1 (1.00–1.41)	1.15±0.08 (0.99–1.31)	0.63
PT-INR	1.07±0.01 (1.05–1.09)	1.09±0.02 (1.04–1.13)	1.07±0.02 (1.04–1.10)	0.54
APTT-T (s)	33.1±0.4 (32.4–33.8)	33.4±0.6 (32.2–34.6)	33.3±0.5 (32.3–34.2)	0.88

*Optimal PCR was defined as ≥118% by ROC analysis. Continuous data are shown as mean±standard error and categorical data as numbers (%). FEV1.0%: forced expiratory volume % in 1 s; SAPT: single antiplatelet therapy; DAPT: dual antiplatelet therapy; PT-INR: prothrombin time-international normalized ratio; APTT-T: activated partial thromboplastin time; AAA: abdominal aortic aneurysm; PCR: platelet count recovery

**Table 2:** Table 2 Characteristics of AAAs and treatment in the patients with and without optimal platelet count recovery

Variables	Total (n=205)	PCR(+) (n=79)	PCR(−) (n=126)	P-value
Abdominal aortic aneurysm				
Size (mm)	5.1±0.5 (5.0–5.3)	5.3±0.9 (5.1–5.4)	5.1±0.7 (4.9–5.2)	0.08
Shape (saccular)	19 (9)	7 (9)	12 (10)	1.00
Stent graft model				
Zenith	60 (29)	30 (38)	30 (24)	0.30
Powerlink	19 (9)	7 (9)	12 (10)	
Excluder	56 (27)	19 (24)	37 (29)	
Endurant II	66 (32)	22 (28)	44 (35)	
Aorfix	4 (2)	1 (1)	3 (2)	
Proximal landing				
Aortic neck length (mm)	26.6±0.8 (25.0–28.2)	26.8±1.4 (24.1–29.6)	26.0±1.1 (23.9–28.2)	0.65
Aortic neck diameter (mm)	21.5±0.2 (21.1–22.0)	22.1±0.4 (21.3–22.8)	21.2±0.3 (20.6–21.8)	0.09
Aortic neck angle (<60°)	177 (86)	68 (86)	109 (87)	0.83
Distal landing				
Bil. common iliac artery landing	180 (88)	69 (87)	111 (88)	1.00
External iliac artery landing	16 (8)	6 (8)	10 (8)	
Bil. external iliac artery landing	2 (1)	1 (1)	1 (1)	
Others	7 (3)	3 (3)	4 (3)	
Operation time (min)	163±4 (155–171)	160±7 (145–174)	167±6 (155–178)	0.46
Blood loss (cc)	303±24 (255–351)	262±43 (177–348)	337±34 (270–405)	0.17
Blood transfusion				
Red blood cells	31 (15)	4 (5)	27 (21)	0.001
Platelets	2 (1)	1 (1)	1 (1)	1.00
Fresh frozen plasma	3 (1)	0 (0)	3 (2)	0.29

Bil.: bilateral. Other abbreviations defined in Table 1.

Sex and smoking history differed significantly between PCR(−) and PCR(+) groups. Preoperative platelet count was also slightly but significantly lower in the PCR(+) group compared with the PCR(−) group (p=0.01) (**Table 1**). Mean size and the prevalence of saccular-shaped aneurysms did not differ between the groups. The EVAR treatment variables stent-graft model, details of the proximal neck and distal landing iliac arteries, and operating time also did not differ between the groups. Alternatively, more PCR(−) patients received red blood cell transfusions than PCR(+) patients (p=0.001).

### Overall survival and cumulative aneurysm sac expansion rates

**Figure 3:**
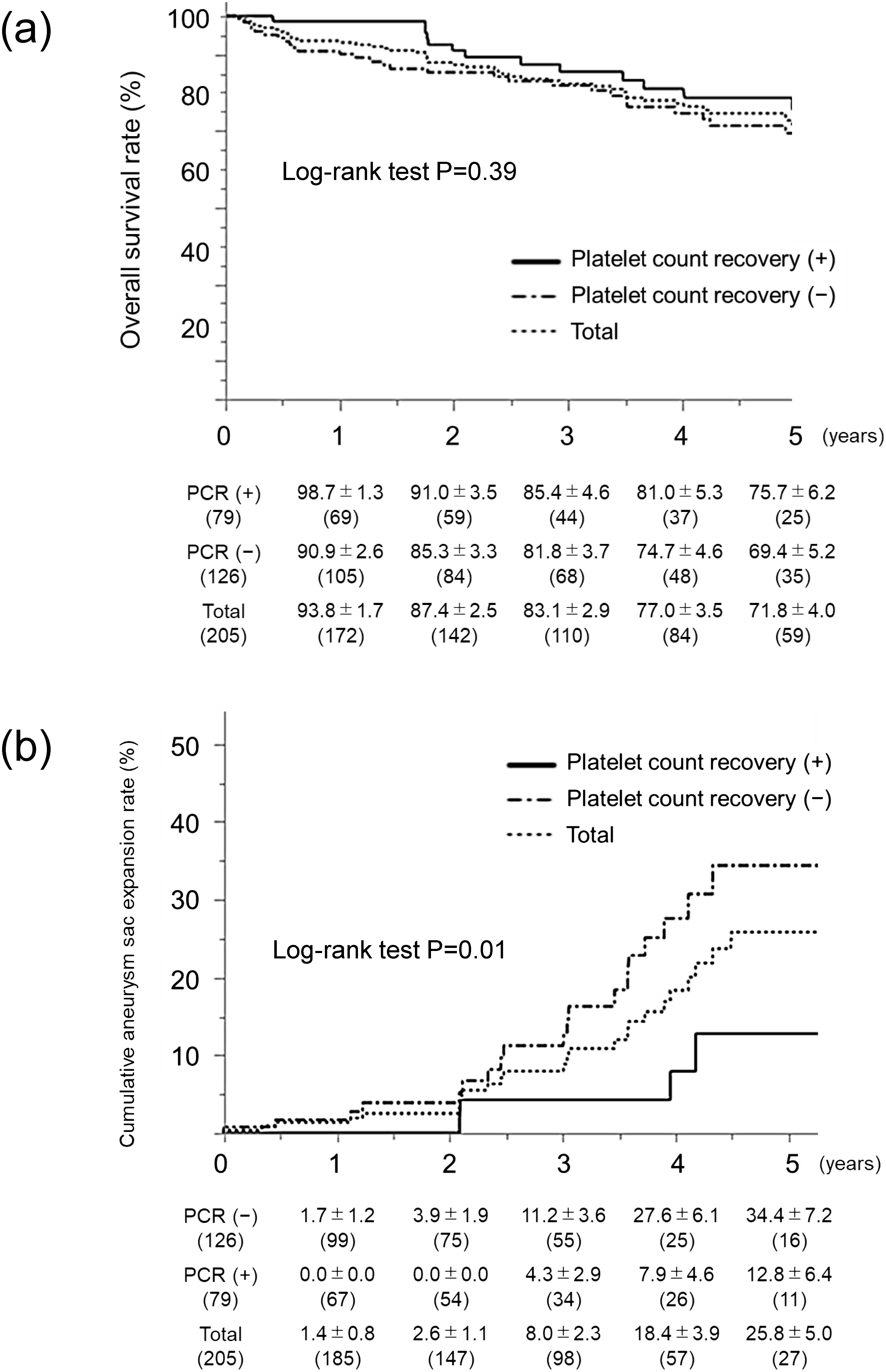
Fig. 3 (**a**) Overall survival rates in patient groups with and without optimal platelet count recovery (PCR, defined as ≥118% of pre-EVAR baseline). (**b**) Cumulative aneurysm sac expansion rates in patient groups with and without optimal PCR. The number at risk is given in parentheses. Continuous data are shown as mean±standard error.

Overall survival rates did not differ between PCR(+) and PCR(−) groups over 5 years post-EVAR (**Fig. 3a**). On the other hand, the cumulative aneurysm sac expansion rate (% of patients) was significantly higher in the PCR(−) than the PCR(+) group (34.4% vs. 12.8% at 5 years, p=0.01) (**Fig. 3b**). Results of univariate and multivariate Cox proportional-hazard analyses of factors related to cumulative aneurysm sac expansion rate are summarized in **Table 3** and in the Supplemental Table. Univariate analysis revealed age (>80 years), female sex, dual antiplatelet therapy (DAPT), lack of PCR (PCR(−) status), EL, and a short aortic neck (<10 mm) as risk factors of sac expansion. Multivariate analysis identified age, EL, and short aortic neck (<10 mm) as significant independent predictors.

EVAR: endovascular aneurysm repair

**Table 3:** Table 3 Multivariate analysis of factors related to cumulative aneurysm sac expansion rate

Variables	Univariate			Multivariate		
	HR	95%CI	P value	HR	95%CI	P value
Characteristics and comorbidities						
Age (>80 years)	3.00	1.36–6.55	0.007	2.82	1.21–6.59	.0169
Sex (female)	3.67	1.61–8.00	<0.01	2.10	0.85–4.99	.1050
Medication						
Antiplatelet therapy			0.04			.0284
SAPT vs. none	1.66	0.70–4.07	0.08	1.49	0.59–3.95	.4030
DAPT vs. none	4.60	1.40–13.46	0.01	5.92	1.67–19.59	.0076
DAPT vs. SAPT	2.77	0.67–7.59	0.08	3.98	1.17–12.36	.0290
Laboratory data						
Disturbed PCR	3.68	1.39–12.66	0.007	3.68	1.28–13.50	.0141
Aneurysms and treatments						
Aortic neck length (<10 mm)	5.42	1.56–14.49	0.01	12.51	3.20–41.98	.0009
Endoleak	107.36	14.52–794.09	<0.0001	97.5	12.36–769.61	<0.0001

HR: hazard ratio; CI: confidence interval. Other abbreviations are the same as in Table 1.

## Discussion

Several studies have documented reduced platelet counts in the days following EVAR.^14,15)^ This response is also accompanied by changes in white blood cell count and C-reactive protein, which are well-known inflammation markers, suggesting that reduced platelet count is due to a postoperative inflammatory response (termed ‘postimplantation syndrome’). Generally, platelet count decreases from roughly 190–200×10^3^/µL before surgery to 150–160×10^3^/µL on POD1 to POD3.^13–15)^ This change is change consistent with the 20%–30% decrease observed 24 and 72 h post-EVAR in the current study. Platelet count then rose, frequently above presurgical baseline, on POD7. These results suggest that platelet count is chronically suppressed in AAA patients and reversed after EVAR delay.

This chronic reduction may stem directly from blood coagulation disorder, causing the aortic aneurysm, as disseminated intravascular coagulation (DIC) has occurred in 4% of AAA patients.^16)^ The aneurysm-induced DIC is suggested to be caused by a hemostatic imbalance due to exposure of the denuded aortic endothelial surface and regulatory dysfunction following the permanent triggering of’ coagulation factors.^17)^ Therefore, a reduced platelet count is an important and relatively sensitive (although not specific) sign of DIC.^18,19)^ In contrast to platelet count, Yamazumi et al. reported that hemostatic factors, such as thrombin–antithrombin III complex (TAT), D-dimer and fibrinogen/fibrin degradation products, were significantly higher in AAA patients than healthy controls.^20)^ They also reported that the values of these hemostatic factors improved but were not completely normalized by EVAR. In contrast, only below-normal platelet counts were significantly increased among AAA patients three months after open repair. These findings collectively indicate that almost all AAA patients suffer more or less from chronic DIC or coagulopathy. Then, platelet count reduction due to AAA recovers soon after successful EVAR, curing the coagulation dysfunction with a complete sealing of the aneurysmal aortic wall. Furthermore, the postoperative inflammatory response contributing to platelet count reduction and the platelet activation indicated by biomarker expression (CD62P-CD36) were correlated with the volume of new-onset thrombus in AAA with AAA volume.^13,14)^ Hence, platelet abundance and function rather than hemostatic factors appear to be more sensitive biomarkers for EVAR success and AAA prognosis.

In this study, PCR at POD7 was negatively influenced by EL, as patients showing persistent EL also exhibited significantly attenuated PCR. Further, a poor PCR was a risk factor for aneurysm sac expansion following EVAR in our univariate analysis (**Table 3**). Moreover, the multivariate analysis disclosed age, EL, and short aortic neck (<10 mm) as independent risk factors. These results are in line with previous studies, which identified age, anatomical condition of aortic neck, and antiplatelet therapy as risk factors for aneurysm sac expansion after EVAR.^1,5,21–23)^ Aortic short neck is a risk factor of type Ia endoleak, which brings massive blood flow to the aneurysm sac and can cause rapid sac expansion. In our analysis, EL, a suggested risk of aneurysm sac expansion, included type I and III ELs. Hence, these results might reflect that type I and III ELs were the most important risk factors of EVAR failure with aneurysm sac expansion. In the real world, however, most type I and III ELs are easily detected with the standard follow-up examinations using CT and/or ultrasound imaging.^9,10)^ Therefore, the remaining issue is the prediction and early detection of delayed and late aneurysm sac expansion. This type of sac expansion was believed to be caused mainly by type II EL. On the other hand, “occult” type I or III EL is suggested to account for more than 20% of type II ELs with treatment failure and/or covert rapid aneurysm sac growth.^24)^ In some difficult cases, an accurate diagnosis of the type of EL relies entirely on imaging with an enhanced medium such as enhanced CT and angiography.^25)^ This fact is inconvenient for AAA patients because their renal function is known to decline during follow-up after EVAR.^26)^ In this study, we revealed that disturbed PCR, which can be revealed by a simple blood test, is associated with type-II EL with aneurysm sac expansion as well as type I and III ELs (**Fig. 2**). This result implies that the serological approach and imaging-based examinations can be a useful and less invasive option to improve postprocedural surveillance accuracy after EVAR.

This study has several limitations. This was a retrospective single-center study with limited sample numbers, which may introduce selection bias and limit the statistical power for the identification of additional risk factors. Second, PCR was based on the CBC test, which is inexpensive, but only an indirect estimation of platelet count. Also, the platelet count can be affected by other systemic conditions, such as hypervolemia and hypovolaemia, as well as by AAA. Finally, the retrospective study design precludes the investigation of causal relationships among PCR, EL, and aneurysm sac expansion. Further studies are required for the optimization of EVAR follow-up, such as using platelet activity-specific markers.

## Conclusion

Platelet count shows the temporal ‘dip’ reduction during POD1–3 followed by recovery up to 112% on POD7 after EVAR. ELs can attenuate the PCR, and the disturbed PCR may be associated with aneurysm sac expansion after EVAR.
