# Supplementary material for: Platelet Count Recovery after Endovascular Aneurysm Repair for Abdominal Aortic Aneurysm
Source: Ann Vasc Dis. 2021 Mar 25;14(1):11–8. doi: 10.3400/avd.oa.20-00030 (PMC7991710; doi:10.3400/avd.oa.20-00030)
Supplement: Supplementary Data [file avd-14-1-oa.20-00030_s001.pdf]

Supplemental Table. Univariate analysis of factors related to cumulative aneurysm sac expansion rate

Variables	Univariate		
	**HR**	**95%CI**	**P-value**
**Characteristics and comorbidities**			
Hypertension	1.21	0.49–3.64	0.69
Diabetes mellitus	1.67	0.71–3.69	0.23
Dyslipidaemia	1.11	0.45–2.47	0.81
Coronary artery disease	1.89	0.86–4.10	0.11
Cerebrovascular disease	0.76	0.28–1.80	0.56
Atrial fibrillation	2.80	0.66–8.18	0.14
End-stage renal disease	7.20	0.39–39.73	0.14
**Medication**			
Statin use	1.48	0.68–3.40	0.32
Anticoagulant therapy	1.87	0.44–5.38	0.35
**Laboratory data**			
Preoperative platelet count (10^3/μL)	0.99	0.99–1.00	0.17
Platelet count on POD1 (10^3/μL)	0.99	0.98 - 1.00	0.02

Platelet count on POD3 (10^3/μL)	0.99	0.98 - 1.00	0.02
Platelet count on POD7 (10^3/μL)	0.99	0.98 - 1.00	0.0009
CRP (mg/dL)	1.08	0.84–1.26	0.47
PT-INR	0.67	0.01–5.57	0.77
APTT-T (s)	0.95	0.86–1.03	0.26
**Aneurysms and treatments**			
Size (>55 mm)	1.07	0.46–2.36	0.86
Shape (saccular)	1.61	0.38–4.68	0.47
Aortic neck diameter (>28 mm)	2.49	0.40–8.43	0.66
Aortic neck angle (>60°)	0.73	0.17–2.11	0.59
Operation time (min)	1.00	0.99–1.00	0.32
Blood loss (cc)	1.00	1.00–1.00	0.35
Blood transfusion	2.18	0.80–5.15	0.12

HR, hazard ratio; CI, confidence interval;

Other abbreviations are the same as in Table 1.
